# Association of Genes, Pathways, and Haplogroups of the Mitochondrial Genome with the Risk of Colorectal Cancer: The Multiethnic Cohort

**DOI:** 10.1371/journal.pone.0136796

**Published:** 2015-09-04

**Authors:** Yuqing Li, Kenneth B. Beckman, Christian Caberto, Remi Kazma, Annette Lum-Jones, Christopher A. Haiman, Loïc Le Marchand, Daniel O. Stram, Richa Saxena, Iona Cheng

**Affiliations:** 1 Cancer Prevention Institute of California, Fremont, California, United States of America; 2 Stanford Cancer Institute, Palo Alto, California, United States of America; 3 University of Minnesota Genomics Center, Minneapolis, Minnesota, United States of America; 4 Epidemiology Program, University of Hawaii Cancer Center, University of Hawaii, Honolulu, Hawaii, United States of America; 5 Centre National de Génotypage, CEA, Evry, France; 6 Biomarker Development, Novartis Institutes for BioMedical Research, Basel, Switzerland; 7 Department of Preventive Medicine, Keck School of Medicine, University of Southern California, Los Angeles, California, United States of America; 8 Center for Human Genetic Research, Department of Anesthesia, Critical Care and Pain Medicine, Massachusetts General Hospital, Boston, Massachusetts, United States of America; 9 Program of Medical and Population Genetics, The Broad Institute of Harvard and MIT, Cambridge, Massachusetts, United States of America; Innsbruck Medical University, AUSTRIA

## Abstract

The mitochondrial genome encodes for the synthesis of 13 proteins that are essential for the oxidative phosphorylation (OXPHOS) system. Inherited variation in mitochondrial genes may influence cancer development through changes in mitochondrial proteins, altering the OXPHOS process, and promoting the production of reactive oxidative species. To investigate the role of the OXPHOS pathway and mitochondrial genes in colorectal cancer (CRC) risk, we tested 185 mitochondrial SNPs (mtSNPs), located in 13 genes that comprise four complexes of the OXPHOS pathway and mtSNP groupings for rRNA and tRNA, in 2,453 colorectal cancer cases and 11,930 controls from the Multiethnic Cohort Study. Using the sequence kernel association test, we examined the collective set of 185 mtSNPs, as well as subsets of mtSNPs grouped by mitochondrial pathways, complexes, and genes, adjusting for age, sex, principal components of global ancestry, and self-reported maternal race/ethnicity. We also tested for haplogroup associations using unconditional logistic regression, adjusting for the same covariates. Stratified analyses were conducted by self-reported maternal race/ethnicity. In European Americans, a global test of all genetic variants of the mitochondrial genome identified an association with CRC risk (P = 0.04). In mtSNP-subset analysis, the NADH dehydrogenase 2 (*MT-ND2*) gene in Complex I was associated with CRC risk at a P-value of 0.001 (q = 0.015). In addition, haplogroup T was associated with CRC risk (OR = 1.66, 95% CI: 1.19–2.33, P = 0.003). No significant mitochondrial pathway and gene associations were observed in the remaining four racial/ethnic groups—African Americans, Asian Americans, Latinos, and Native Hawaiians. In summary, our findings suggest that variations in the mitochondrial genome and particularly in the *MT-ND2* gene may play a role in CRC risk among European Americans, but not in other maternal racial/ethnic groups. Further replication is warranted and future studies should evaluate the contribution of mitochondrial proteins encoded by both the nuclear and mitochondrial genomes to CRC risk.

## Introduction

Colorectal cancer is the third most common cancer among men and women in the United States. In 2015, an estimated 220,800 new colorectal cancers (CRC) were diagnosed in the United States [1]. Approximately 35% of the risk of CRC is attributed to inherited factors [2]. Close to fifty risk loci for CRC have been identified by genome-wide association studies, which have focused on common variants of the nuclear genome [3–7]. However, these loci explain only a small proportion of the heritability of colorectal cancer and additional heritable factors remain to be discovered.

Over 70 years ago, Otto Warburg reported an altered metabolism among cancer cells characterized by an increase in glucose uptake and glycolysis despite an adequate oxygen supply for mitochondrial respiration, a phenomenon referred as ‘aerobic glycolysis’.[8] Warburg hypothesized that this shift towards ‘aerobic glycolysis’ signified a deficiency in mitochondrial respiration, representing a fundamental cause of cancer.[9] This observation has now been confirmed in many types of cancer cells that exhibit elevated levels of glucose transport and increased rates of glycolysis—referred to as the Warburg effect.[10, 11]

The mitochondrial genome is a double-stranded circular DNA molecule of 16,569 base pairs which is highly polymorphic and contains almost no intergenic regions. [12, 13] The proteins it encodes for essential functions in cellular metabolism and regulation of cell death[14]. Thirty-seven proteins are encoded by the mitochondrial DNA (mtDNA), of which 13 are involved in the oxidative phosphorylation (OXPHOS) machinery and 24 make up the RNA machinery (2 ribosomal RNAs and 22 transfer RNAs). The primary function of the mitochondrion is the production of the energy molecule, adenosine triphosphate (ATP), through the metabolic OXPHOS pathway.

Variations in mtDNA, including mitochondrial single nucleotide polymorphisms (mtSNPs), have the potential to modify mitochondrial function and lead to increased oxidative stress and cancer risk [15–17]. A Scottish study examined 132 mtSNPs in 2,854 cases and 2,822 controls and found no association with overall CRC risk. [18]. To our knowledge, no study to date has comprehensively examined the relationship between mtDNA variants and CRC risk across different racial/ethnic populations. Furthermore, a pathway based approach, which increases study efficiency for effects of modest size, may help to reveal associations between the mitochondrial genome and cancer risk.

Mitochondrial haplogroups are defined by unique sets of mtSNPs, reflecting specific ancestral populations as a result of the sequential accumulation of mitochondrial mutations through maternal lineages. Mitochondrial haplogroups have been associated with breast, prostate, and nasopharyngeal cancers [19–22]. Three studies have investigated the association between mitochondrial haplogroups and CRC risk in European and Asian populations with inconsistent results [18, 21, 23].

To comprehensively examine the role of the mitochondrial genome and CRC risk across multiple racial/ethnic groups, we genotyped a set of 185 mtSNPs to evaluate the association of genetic variation in the mitochondrial genome, pathways and genes, as well as of single mtSNPs and haplogroups, among 2,453 CRC cases and 11,930 controls of the Multiethnic Cohort (MEC) Study.

## Materials and Methods

### Study Subjects

The MEC is a large population-based cohort study of more than 215,000 men and women from Hawaii and California. The cohort is predominantly comprised of individuals from five racial/ethnic groups: African Americans, Asian Americans, European Americans, Latinos, and Native Hawaiians. Participants between the ages of 45 and 75 years were recruited from March 1993 through May 1996 and completed a 26-page self-administered questionnaire that included information regarding medical history, family history of cancer, diet, dietary supplements, medication use, and physical activity. Further details about this cohort are provided elsewhere [24].

Incident CRC cases were identified up to December 9, 2010 by cohort linkage to population-based Surveillance, Epidemiology and End Results (SEER) cancer registries covering Hawaii and California. Information on stage of disease at the time of diagnosis was also collected from the cancer registries. Blood samples were collected from incident colorectal, breast, and prostate cancer cases after their diagnosis, as well as a random sample of cohort members to serve as controls from 1996 through 2001, and prospectively from all willing surviving participants from 2002 through 2007. Informed consent was obtained at blood draw. Among the CRC cases used in this analysis, 70.4% had their blood drawn after diagnosis and 29.6% prior to diagnosis. Control subjects were men and women selected to serve as matched controls for nested case-control studies of colorectal, breast and prostate cancer. They were also selected to not have developed CRC before cohort entry or during follow-up as of December 9, 2010. This nested case-control study consisted of 2,453 CRC cases and 11,930 controls.

This study was approved by the institutional review board at the Cancer Prevention Institute of California.

### mtSNP selection and genotyping

We abstracted mtSNP information from publicly deposited mtDNA sequencing data (PhyloTree mtDNA build 8, March 21, 2010) for 3,674 individuals comprising 599, 1,401, 1,118 and 556 subjects of African, European, Asian, and Latino ancestry, respectively. In addition, we sequenced the mtDNA of 160 Native Hawaiians using the Affymetrix resequencing array and identified 241 mtSNPs (MAF > 2%) in this population at a density of 1 mtSNP per 64 base pairs with an average call rate of 90.6% [25]. A total of 863 mtSNPs were selected, including 160 mtSNPs identified from the sequencing data and all missense mtSNPs (n = 230) and those previous associated with cancer (n = 37).

The genotyping of mtDNA was carried out in three phases using the Sequenom MassArray platform (Sequenom, San Diego). In the phase I, quantitative allelotyping was performed on DNA pools from 75 samples, to enable the rapid and affordable screening of the entire list of 863 putative mtSNPs. Allelotyping provides a quantitative estimate of allelic frequency in a mixture of DNA [26] with the goal of phase I to eliminate those mtSNPs with an undetectable minor allele frequency (MAF). A total of 240 mtSNPs were eliminated in phase I. In phase II, 619 of the remaining 623 mtSNPs were genotyped in a multiethnic panel of 376 subjects using the Sequenom iPLEX platform, providing robust MAFs of these mtSNPs across all five major ethnicities. Of the 619 mtSNPs genotyped, 186 mtSNPs were identified to have MAF greater than 0.02. In phase III, these 186 mtSNPs were genotyped in our nested CRC case-control study of 2,498 cases and 12,070 controls, using the Sequenom iPLEX platform. A total of 185 mtSNPs passed our quality control criteria of 95% call rate and MAF threshold >0.001. Stratifying on reported maternal race/ethnicity, 175, 168, 165, and 102 mtSNPs had a MAF>0.001 in African Americans, European Americans, Latinos, and Asian Americans, respectively; and 50 mtSNPs had a MAF > 0.005 in Native Hawaiians (using a less stringent threshold due to smaller sample size) (S1 Table). A total of 2,453 CRC cases and 11,930 controls were successfully genotyped with a call rate > 95%. The average individual call rate was 99.6% and the average concordance rate for 8% replicated samples was 99.7%.

### Statistical analysis

To evaluate the cumulative effect of all mtDNA variants, variants in the OXPHOS pathway, complexes, and genes, we used the sequence kernel association test (SKAT_commonrare) [27–29]. The SKAT_commonrare test is an omnibus procedure allowing for both rare and common variants to contribute to the overall test statistic [29]. To estimate haplogroups, we used the HaploGrep software (http://www.haplogrep.uibk.ac.at) based on Phylotree build 16 [30, 31] and categorized individuals based on the major haplogroups. We conducted unconditional logistic regression to examine the association between major haplogroups and CRC risk, using the most common haplogroup as the reference category. To test for single mtSNP associations with CRC risk, we also conducted unconditional logistic regression estimating p-values using a 1-degree-of-freedom Wald test. The overall analysis was adjusted for age, sex, self-reported maternal race/ethnicity, and the first five principal components of global ancestry. Principal components of genetic ancestry were estimated from genotype data for a panel of 128 ancestry informative markers genotyped in the MEC [32, 33]. Previous work in the Multiethnic Cohort has shown that modest population stratification within simulated nested case-control studies was readily corrected for by adjusting for race/ethnicity or the top principal components of ancestry [34]. Additional adjustment for family history of colorectal cancer, dietary intakes of fiber, calcium, folate, alcohol, vigorous physical activity, and smoking did not notably alter results. Thus, these covariates were not included in our final multivariate models. Moreover, we also tested all these associations stratifying on self-reported maternal race/ethnicity and anatomical subsite. All statistical tests presented are two-sided. A false discovery rate (FDR) was estimated to address p-value inflation due to multiple hypothesis testing and a q value<0.1 was used to determine statistical significance.

**Fig 1:**
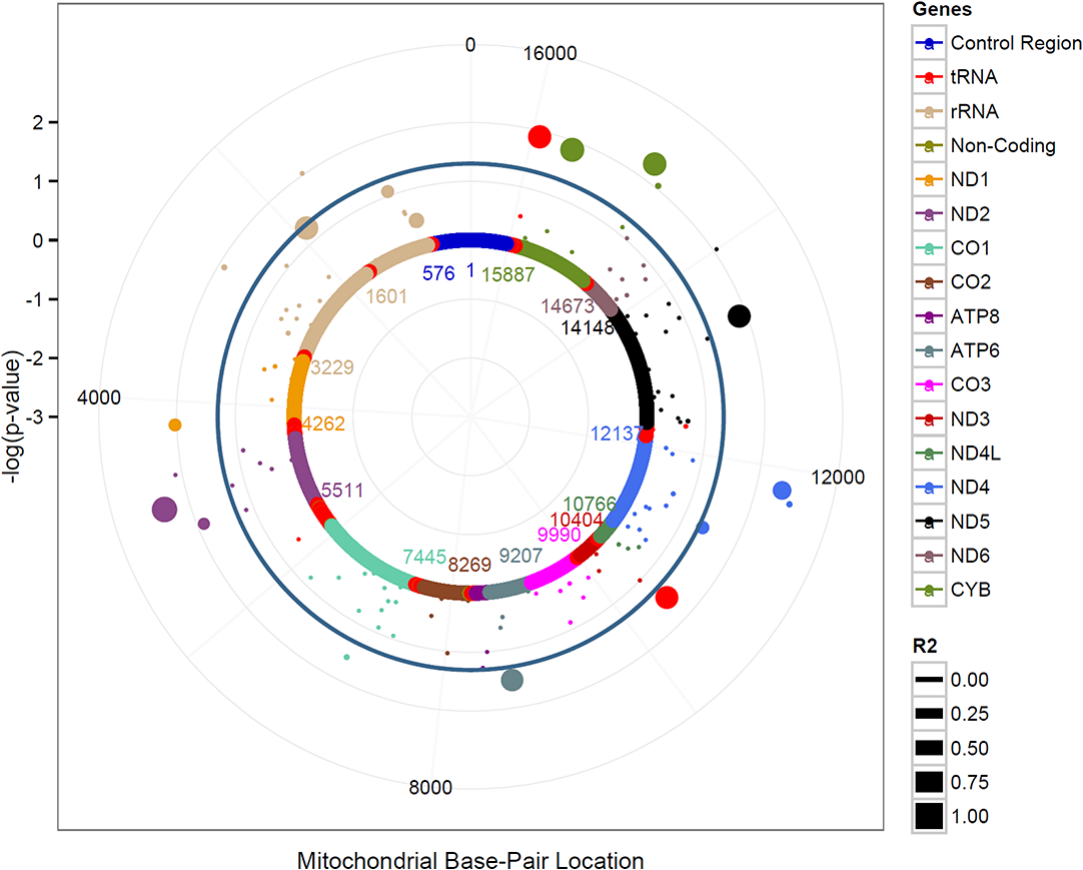
Mitochondrial solar plot for European Americans. From outside to inside, the three grey circles correspond to the P value of 10^–3^, 10^–2^and 10^–1^. The teal circle represents a p-value of 0.05. Each dot represents the mtSNP association with CRC color coded by mitochondrial gene. The size of each dot represents the correlation (R2) between mt4917 and other mtSNPs among European Americans.

Single mtSNP analyses were done using PLINK software (version 1.9). mtSNP-set based analyses were done using the SKAT package in R (version 3.0.3). The Mitochondrial solar plot (Fig 1) was drawn using ggplot2 package in R.

## Results

### Study Characteristics

Study characteristics of the 14,383 study subjects (2,453 CRC cases; 11,930 controls) are presented in Table 1. Colorectal cases were older and had a higher proportion of males than controls. The distribution of self reported maternal race/ethnicity included Asian Americans (28.69%), African Americans (24.35%), European Americans (21.42%), Latinos (20.45%), and Native Hawaiians (4.90%). Cases were more likely to report a family history of colorectal cancer, a history of polyps, and a history of diabetes than controls. Approximately 76% of cases occurred in the colon and 50% of cases were localized stage.

**Table 1:** Study characteristics of 2,453 colorectal cancer cases and 11,930 controls.

Characteristics	Categories	Cases (n = 2,453)	Controls (n = 11,930)
Age, mean (SD)		70.4 (8.34)	68.5 (8.39)
Sex, n (%)	Male	1363 (55.56%)	6674 (55.94%)
	Female	1090 (44.44%)	5256 (44.06%)
Maternal race/ethncity, n(%)*	African Americans	464 (18.83%)	3025 (25.36%)
	Asian Americans	809 (32.98%)	3307 (27.72%)
	Latinos	577 (23.52%)	2361 (19.81%)
	European Americans	482 (19.65%)	2581 (21.63%)
	Native Hawaiians	113 (4.61%)	594 (4.98%)
BMI, n (%)	<25 kg/m2	775 (37.17%)	4025 (37.62%)
	> = 25 kg/m2	1310 (62.83%)	6675 (62.38%)
Family history of colorectal cancer, n (%)	Yes	272 (11.09%)	1045 (8.83%)
History of polyp of intestine, n (%)	Yes	307 (12.52%)	1078 (9.04%)
History of Diabetes, n (%)	Yes	388 (15.19%)	1515 (12.70%)
Pevious colonoscopy/sigmoidoscopy, n (%)	Yes	1128 (45.98%)	4877 (40.88%)
Dietary Intake, mean (SD)	Fiber, g/kcal/day	11.82 (4.49)	11.92 (4.26)
	Calcium, mg/day	969.94(583.38)	1010.25 (640.29)
	Folate, mcg/day	532.24 (355.76)	541.26 (363.47)
	Alcohol, g/day	11.42 (30.95)	9.31 (24.59)
Vigorous physical acitiviy, hours/day, mean (SD)		0.35 (0.79)	0.39 (0.79)
Smoking, pack/year, mean (SD)		11.98 (16.29)	10.38 (14.75)
Site, n (%)*	Colon	1870 (76.23%)	
	Rectal	536 (21.85%)	
Stage, n (%)*	Localized	1221 (49.78)	
	Advanced	1128 (45.98)	

*Due to missing value, the total doesn't add up to 100%.

### Mitochondrial Genome, Pathway, and Gene Associations

A global test of all 168 mtSNPs in the mitochondrial genome (MAF >0.001) showed a significant association with CRC risk in self-reported maternal European Americans (P = 0.04; Table 2), while no associations were seen in other maternal racial/ethnic groups or in the whole sample (S2 Table). For European Americans, when restricting the mtSNP-set to the OXPHOS pathway, comprised of 133 mtSNPs, the association with CRC risk had a P = 0.029 (q = 0.054 Table 2). Within the OXPHOS pathway, complex I (80 mtSNPs; P = 0.025; q = 0.081) and complex III (15mtSNPs; P = 0.027; q = 0.081) were associated with CRC risk. To further investigate the Complex I association, we conducted an analysis focusing on missense and non-missense mtSNPs separately. Collectively, both missense (22 mtSNPs, P = 0.024) and non-missense mtSNPs (58 mtSNPs, P = 0.04) in Complex I were associated with CRC risk at P<0.05 (S3 Table).

**Table 2:** Association between mitochondrial genome, pathways, genes and CRC risk in European Americans.

Mitochondrial genome^#^		Mitochondrial Oxidative phosphoralation pathway^#^			Mitochondrial complexes^#^				Mitochondrial genes^#^			
No. of mtSNPs	P value	No. of mtSNPs	P value	q value^*^	Complex	No. of mtSNPs	P value	q value^*^	Gene	No. of mtSNPs	P value	q value^*^
**168**	**0.040**	**133**	**0.029**	0.054	**Complex I**	**80**	**0.025**	**0.081**	*MT-ND1*	9	0.24	0.36
									***MT-ND2***	**14**	**0.001**	**0.015**
									*MT-ND3*	6	0.29	0.36
									***MT-ND4***	**16**	**0.015**	0.113
									*MT-ND4L*	3	0.43	0.48
									*MT-ND5*	24	0.15	0.32
									*MT-ND6*	8	0.28	0.36
					**Complex III**	**15**	**0.027**	**0.081**	***MT-CYB***	**15**	**0.027**	0.14
					Complex IV	28	0.198	0.297	*MT-CO1*	14	0.11	0.28
									*MT-CO2*	5	0.48	0.48
									*MT-CO3*	9	0.48	0.48
					Complex V	10	0.095	0.19	*MT-ATP6*	8	0.28	0.36
									***MT-ATP8***	**2**	**0.036**	0.14
									rRNA	27	0.17	0.32
									tRNA	8	0.10	0.28

#Association tests were adjusted for age, sex and global ancestry PC1-PC5

* False Discovery Rate test

Of the thirteen genes and the rRNA and tRNA subunits in the mitochondrial genome, four genes were associated with CRC at a P value < 0.05: mitochondrially encoded NADH dehydrogenase 2 (*MT-ND2*) (P = 1.0x 10^–3^) and mitochondrially encoded NADH dehydrogenase 4 (*MT-ND4*) (P = 0.015) in complex I; mitochondrially encoded cytochrome b (*MT-CYB*) (P = 0.027) in complex III; and mitochondrially encoded ATP synthase 8 (*MT-ATP8*) (P = 0.036) in Complex V. The *MT-ND2* gene remained significantly associated with CRC after multiple correction (q = 0.015; Table 2). Both missense and non-missense mtSNPs in the *MT-ND2* were associated with CRC risk at P<0.05 (2 mtSNPs Pmissense = 0.008, 12 mtSNPs Pnon-missense = 0.006; S3 Table). In a stratified analysis by anatomical subsite (S4 Table), a stronger association for *MT*-*ND2* was seen in colon tumors (P = 7.0x10^–4^) and no association was seen in rectal tumors (P = 0.79).

### mtSNP Associations

Overall, 14 of 185 mtSNPs were associated with CRC at P<0.05 in the total study population (S5 Table). In stratified analysis by maternal race/ethnicity, 7 of 154 mtSNPs, 4 of 97 mtSNPs, 18 of 147 mtSNPs, and 22 of 156 mtSNPs were associated with CRC risk in African Americans, Asian Americans, European Americans, and Latinos, respectively at P<0.05 (S5 Table). No mtSNP associations were observed in Native Hawaiians. Of the 14 mtSNPs associated with overall CRC risk, the most significant association was seen with the missense mtSNP, mt4917 located in *MT-ND2* (OR = 1.52; 95% CI: 1.16–2.01; P = 0.0029, q = 0.308). The minor allele mt4917 (G) varies substantially across the five maternal racial/ethnic groups. Specifically, mt4917 was common in European Americans (MAF = 0.10), rare in African American (MAF = 0.005), Latinos (MAF = 0.006), and was monomorphic in Asian Americans and Native Hawaiians. In European Americans, three mtSNPs in the gene *MT-ND2* were nominally associated with CRC risk at P<0.05 (Table 3). The strongest association was observed with mt4917 (OR = 1.55; 95% CI:1.15–2.10; P = 0.004, q = 0.16). Fig 1 presents the mitochondrial solar plot (given the circular nature of mtDNA) of mtSNP associations with CRC risk among European Americans and the correlation between mtSNPs with mt4917. There was a high correlation between mt4917 (r^2^ >0.75) and the seven other mtSNPs across the mitochondrial genome.

**Table 3:** Association between mtSNPs in MT-ND2 and CRC risk by self-reported maternal race/ethnicity and overall.

		Major Allele	Minor Allele	MAF	OR (95%CI)	P value
mt4917	African Americans	A	G	0.005	0.87 (0.20, 3.84)	0.850
	Asian Americans	A	G	0.001	—	—
	Latinos	A	G	0.006	1.89 (0.71, 5.04)	0.200
	Native Hawaiins	A	G	0.002	—	—
	**European Americans**	**A**	**G**	**0.101**	**1.55 (1.15, 2.10)**	**4.00E-03**
	**Overall**	**A**	**G**	**0.025**	**1.52 (1.16, 2.01)**	**2.90E-03**
mt4655	African Americans	G	A	0.028	1.60 (0.96,2.68)	0.070
	Asian Americans	G	A	0.028	0.86 (0.52,1.43)	0.560
	Latinos	G	A	0.002	—	—
	Native Hawaiins	—	—	—	—	—
	**European Americans**	G	A	**0.001**	**8.67 (1.79,42.06)**	**0.007**
	Overall	G	A	0.016	1.22 (0.86,1.72)	0.26
mt4883	African Americans	C	T	0.002	—	—
	Asian Americans	C	T	0.347	0.95 (0.80,1.12)	0.524
	Latinos	C	T	0.069	0.70 (0.46,1.07)	0.102
	Native Hawaiins	C	T	0.012	—	—
	European Americans	C	T	0.005	2.66 (0.96,7.37)	0.061
	Overall	C	T	0.112	0.91 (0.78,1.06)	0.21
mt5147	African Americans	G	A	0.094	0.98 (0.68,1.39)	0.892
	Asian Americans	G	A	0.054	0.99 (0.69,1.41)	0.938
	Latinos	G	A	0.016	0.33 (0.10,1.08)	0.067
	Native Hawaiins	—	—	—	—	—
	**European Americans**	**G**	**A**	**0.055**	**1.62 (1.11,2.38)**	**0.013**
	Overall	G	A	0.054	1.06 (0.86,1.30)	0.575
mt5178	African Americans	C	A	0.002	—	—
	Asian Americans	C	A	0.348	0.95 (0.80,1.12)	0.539
	Latinos	C	A	0.069	0.70 (0.46,1.07)	0.102
	Native Hawaiins	C	A	0.012	—	—
	European Americans	C	A	0.006	2.52 (0.91,6.93)	0.074
	Overall	C	A	0.112	0.91 (0.78,1.06)	0.216

Log additive model adjusted for age, sex, global ancestry PC1 to PC5 and maternal ethnicity in overall analysis; adjusted for age, sex, global ancestry PC1 to PC5 in each maternal race/ethnicity.

### Haplogroup Associations

Haplogroup T was common in European ancestry populations, occurring at a frequency of 9.6% in controls and absent in the other racial/ethnic groups, was significantly associated with CRC risk in European Americans (OR = 1.66, 95% CI: 1.19–2.33, P = 0.003, Pcorrection = 0.015, Table 4). Haplogroup L was associated with CRC risk (OR = 1.54, 95%CI: 1.02–2.31, P = 0.039, Pcorrection = 0.20) in Latinos (4.8% in controls). No clear associations with haplogroups were observed among the remaining racial/ethnic groups (S6 Table).

**Table 4:** Association between haplogroup and CRC risk in European Americans.

Haplogroup^	Case/Control %	OR (95%CI)	P value*
H	34.02 / 39.91	1.00	
A	1.24 / 1.59	1.21 (0.47, 3.14)	0.69
B	1.45 / 1.51	0.77 (0.25, 2.41)	0.66
HV	3.32 / 4.07	0.89 (0.48, 1.64)	0.72
I	1.04 / 1.51	0.51 (0.15, 1.69)	0.27
J	8.51 / 8.91	1.13 (0.77, 1.68)	0.52
K	9.75 / 8.49	1.33 (0.92, 1.93)	0.13
L	2.70 / 2.67	0.99 (0.47, 2.07)	0.98
N	2.90 / 2.48	1.46 (0.78, 2.73)	0.24
**T**	**14.11 / 9.57**	**1.66 (1.19, 2.33)**	**0.003**
U	14.73 / 13.33	1.31 (0.96, 1.81)	0.09
W	1.66 / 2.17	0.99 (0.46, 2.12)	0.97
X	1.24 / 1.28	1.37 (0.56, 3.35)	0.49
Freq less than 1% bin	3.32 / 2.52	1.61 (0.87, 2.99)	0.13

^Haplogroup estimation was based on HaploGrep (version 2.0) using PhyloTree 16.

*Log additive model was adjusted for age, sex and global ancestry PC1 to PC5.

## Discussion

In this study of 14,383 CRC cases and controls, we comprehensively examined the contribution of the mitochondrial genome to CRC risk. To our knowledge, this is the first study to systematically evaluate the mitochondrial genome and its pathway, gene sets, and haplogroups in relation to CRC across multiple maternal racial/ethnic groups. Pathway analyses revealed that the mitochondrial genome and the oxidative phosphorylation pathway play a suggestive role in the CRC risk among European Americans. In addition, an association between the *MT-ND2* gene and CRC risk was observed among European Americans with stronger association seen in colon tumors. Haplogroup T was found to be associated with CRC risk among European Americans independent of global ancestry.

Our analysis of the entire mitochondrial genome demonstrated evidence of an association with CRC risk in the European Americans (P = 0.04), in which the OXPHOS pathway may play an important role (P = 0.029; q = 0.054). A byproduct of OXPHOS is the production of reactive oxygen species (ROS), which can generate free radicals and is involved in many cellular processes including apoptosis, inflammation and oxidative stress that may contribute to aging, degenerative diseases and cancer [15, 35]. Our gene based analysis further suggested that *MT-ND2*, a member of the OXPHOS pathway that encodes for the subunit of *NADH*, is associated with CRC risk in European Americans (P = 0.001; q = 0.015). A recent study reported over expression of *MT-ND2* in CRC tumors vs. normal tissue, which was correlated with lower methylation of the mtDNA D-loop and also significantly associated with stage of disease [36]. These findings support the role of *MT-ND2* in CRC development.

The distribution of haplogroups in the MEC was consistent with previously published data on U.S. population-based samples [37]. The frequency of haplogroup T among our European American control subjects (9.57%) is consistent with the Mitomap database (variance between 8%-11% from West to East Europeans) [38] and non-Hispanic Whites in the National Health and Nutrition Examination Surveys (NHANES) (9.6%)[37]. Two studies have reported no associations between mtDNA haplogroups and CRC risk among Chinese and Scottish populations [18, 21], while an association between haplogroup B4 and CRC risk was reported in a Korean population [23]. We identified an association between haplogroup T and CRC risk in European Americans independent of global ancestry. Haplogroup T is defined by nine polymorphisms [30, 39], including five RNA variants (G709A, G1888A, T8697A, T10463C, G15928A), three synonymous (G13368A, G14905A, A15607G), and one non synonymous (A4917G) polymorphisms. The mtSNP A4917G is the diagnostic mtSNP for haplogroup T and a highly conserved polymorphism in the *MT-ND2* gene [22, 30, 39]. The lack of an association with haplogroup T in the Scottish study [18] may be due to the use of different mtSNPs to define haplogroup T (T4217, G10399A and A12309G). Ruiz-Pesini et al. [22]hypothesized that mt4917 has been retained by adaptive selection and is believed to play an important role in human migration out of Africa into colder climates, with only the *MT-ND2* lineage retrained in haplogroup T due to selection pressures [22]. This may explain the higher frequency of mt4917 in European Americans and its relative absence in African Americans, Latinos, Asian Americans, and Native Hawaiians.

The Scottish study found no association between 132 mtSNPs and overall CRC risk, yet suggested the variant A5657G in *tRNA* (MAF = 0.01) was associated with colon tumors (P = 0.002) [18]. While we did not genotype this mtSNP, which is located close to the *MT-ND2* gene (145 base pair distance), we did observe an association between the *MT-ND2* gene and colon tumors (P = 7.0x10^–4^), which may support the reported association. Given the wide spectrum of risk alleles of rare, low-frequency, and common genetic variants in mitochondrial genome, our study is strengthened by using the SKAT common/rare approach to collectively test multiple risk alleles that may have modest effects [27, 28]. This approach has improved power compared to single SNP tests in the presence of correlation between SNPs and overcomes the limitation of previous methods that upweight rare variants [29, 40]. Using this approach, we were able to capture the role of *MT-ND2* gene and CRC risk. In addition, our study strengths include the investigation of multiple racial/ethnic populations, the examination of mtSNPs based on sequencing data for all five populations, and a comprehensive evaluation of the mitochondrial genome and CRC risk. Limitations of this study include the modest sample size for each population to detect weak genetic effects, particularly among Native Hawaiians. For a mtSNP with MAF = 0.10 and alpha = 0.05, our study has 80% power to detect a minimum OR of 1.40 in African Americans, OR = 1.32 for Japanese Americans, OR = 1.38 for Latinos, OR = 1.40 for European Americans, and OR = 1.80 for Native Hawaiians. In addition, there is a possibility of false positive results given the number of hypothesis tested as our findings do not meet a stringent Bonferroni correction.

In summary, our study suggests that variation in the mitochondrial genome may play a role in CRC risk among European Americans. The findings of associations between genetic variants in *MT-ND2* and haplogroup T with CRC risk warrants replication in other European American populations. Future studies should examine the expression of *MT-ND2* in colorectal tumor and test mitochondrial genes encoded by both the nuclear and mitochondrial genomes to fully examine their contribution to CRC risk.

## Supporting Information

S1 Table 185 mtSNPs tested in 2,453 colorectal cancer cases and 11,930 controls. (XLSX)

S2 Table Association between mitochondrial genome, pathways, genes and CRC risk by self-reported maternal race/ethnicity and overall. (XLSX)

S3 Table mtDNA complex and gene based models of CRC risk in European Americans. (XLSX)

S4 Table Association between mitochondrial genome, pathways, genes and colon and rectal cancer in European Americans. (XLSX)

S5 Table Association between 185 mtSNPs and CRC risk overall and by self-reported maternal race/ethnicity. (XLSX)

S6 Table Association between mitochondrial haplogroups and colorectal cancer risk by self-reported maternal race/ethnicity and overall. (XLSX)
